# Relationship between Amyloid-β Deposition and the Coupling between Structural and Functional Brain Networks in Patients with Mild Cognitive Impairment and Alzheimer’s Disease

**DOI:** 10.3390/brainsci11111535

**Published:** 2021-11-19

**Authors:** Hui Zhang, Edward S. Hui, Peng Cao, Henry K. F. Mak

**Affiliations:** 1 Department of Diagnostic Radiology, The University of Hong Kong, Hong Kong 999077, China; shirlzh7@hku.hk (H.Z.); caopeng1@hku.hk (P.C.); 2 Alzheimer’s Disease Research Network, The University of Hong Kong, Hong Kong 999077, China; 3 Department of Rehabilitation Sciences, The Hong Kong Polytechnic University, Hong Kong 999077, China; 4 State Key Laboratory of Brain and Cognitive Sciences, The University of Hong Kong, Hong Kong 999077, China

**Keywords:** amyloid-β deposition, Alzheimer’s disease, coupling between functional and structural networks, ^18^F-flutemetamol PET-CT, resting-state functional magnetic resonance imaging

## Abstract

Previous studies have demonstrated that the accumulation of amyloid-β (Aβ) pathologies has distinctive stage-specific effects on the structural and functional brain networks along the Alzheimer’s disease (AD) continuum. A more comprehensive account of both types of brain network may provide a better characterization of the stage-specific effects of Aβ pathologies. A potential candidate for this joint characterization is the coupling between the structural and functional brain networks (SC-FC coupling). We therefore investigated the effect of Aβ accumulation on global SC-FC coupling in patients with mild cognitive impairment (MCI), AD, and healthy controls. Patients with MCI were dichotomized according to their level of Aβ pathology seen in ^18^F-flutemetamol PET-CT scans—namely, Aβ-negative and Aβ-positive. Our results show that there was no difference in global SC-FC coupling between different cohorts. During the prodromal AD stage, there was a significant negative correlation between the level of Aβ pathology and the global SC-FC coupling of MCI patients with positive Aβ, but no significant correlation for MCI patients with negative Aβ. During the AD dementia stage, the correlation between Aβ pathology and global SC-FC coupling in patients with AD was positive. Our results suggest that Aβ pathology has distinctive stage-specific effects on global coupling between the structural and functional brain networks along the AD continuum.

## 1. Introduction

Alzheimer’s disease (AD), a chronic disease characterized by progressive memory loss and the deterioration of other cognitive functions, is one of the most common forms of dementia and results in death within 3 to 9 years after diagnosis [1]. Patients with AD are characterized by the progressive accumulation of two neuropathological hallmarks of AD, amyloid-β (Aβ) and tau [2], which eventually lead to the severe impairment of cognition and behavior.

Amyloid-β initially accumulates in the medial frontal and parietal cortices [3,4], while tau accumulates in the medial temporal lobe [5]. These pathological hallmarks of AD follow stage-dependent changes along the AD continuum [6]. It is of note that 82% of mild cognitive impairment (MCI) patients with Carbon-11 Pittsburgh compound B (^11^C-PIB) retention, also known as Aβ-positive, at baseline converted to AD at follow-up [7]; these patients (MCI_Aβ+) also converted to AD faster than MCI patients who were Aβ-negative (MCI_Aβ-) [7,8,9].

Accumulation of Aβ has been shown to be associated with structural changes in subcortical volumes, cortical thickness, and surface area measures in healthy controls (HC) and MCI patients [10]. On the other hand, Aβ and tau pathologies also affect the anatomical and functional connections of the whole brain (known as structural and functional brain networks, respectively) in a stage-dependent manner [11]. It is of note that previous studies have focused on investigating the effect of AD pathologies on the structural or functional brain network alone. Considering these two types of brain connections are tightly coupled, whereby static anatomical connections facilitate and constrain the dynamic functional interactions between different brain regions [12,13,14], whether AD neuropathologies also exhibit stage-dependent effects on the interplay between these two types of brain connections warrants further investigation.

A potential approach to describe the interplay between the structural and functional brain networks is by the correlation between the two networks, known as structure-function (SC-FC) coupling [12,13,14]. The premise is that SC-FC coupling reflects the extent to which the structural brain network is constraining the functional brain network. The higher the coupling, the more the structural brain network constrains the functional brain network [15]. Recent studies have demonstrated that SC-FC coupling is decreased in patients with bipolar disorder [16], Parkinson’s disease (PD) [17], multiple sclerosis (MS) [18], and AD [19]. The spatial variation in SC-FC coupling was found to be consistent with the functional specialization of the cortex; SC-FC coupling of the rostrolateral prefrontal cortex was correlated with executive function in young adults [15].

The central hypothesis of this study is that SC-FC coupling is a novel biomarker for the characterization of the effect of Aβ accumulation along the AD continuum. We have previously shown that the majority of MCI patients can be distinguished from AD patients based on ^18^F-flutemetamol PET-CT scans with high efficacy [20]. Those who could not be differentiated were the MCI_Aβ+ patients who had similar Aβ retention as AD patients. In this study, we therefore aim to investigate whether the effect of Aβ pathology on the global coupling between structural and functional brain networks would differ along the AD continuum, as defined by Aβ retention on ^18^F-flutemetamol PET-CT scans.

## 2. Materials and Methods

### 2.1. Participants

Forty-seven patients older than 55 years-old were recruited from the memory clinic of a university hospital for an MRI and ^18^F-flutemetamol PET-CT scans between June 2017 and June 2019. Subjects with a history of stroke, head injury, seizures, migraine, cancer within five years, active infection, end-stage renal or other organ failures, non-ambulatory or psychiatric diseases, regular alcohol consumption, and drug abuse were excluded. Fourteen healthy adults were recruited from social centers. Inclusion criteria included normal blood pressure (less than 140/90 mmHg) and a normal Montreal Cognitive Assessment (MoCA) cognitive score (≥26).

Ethical approval from the Institutional Review Board of the University of Hong Kong and Hospital Authority Hong Kong West Cluster was obtained (IRB reference number: UW 11-126, 11 April 2015), and our study complied with the Declaration of Helsinki. Written informed consent was obtained from all non-demented participants and the next of kin or caregivers of demented subjects. All participants underwent clinical evaluation, neuropsychological testing, and MRI including structural MRI, resting state functional MRI (rs-fMRI), diffusion tensor imaging (DTI), and arterial spin labelling (ASL). Only patients with mild cognitive impairment (MCI) or Alzheimer’s disease (AD) received magnetic resonance angiography (MRA) and ^18^F-flutemetamol PET-CT scans. The MRI and PET-CT scans were performed within one week of each other.

### 2.2. Clinical and Neuropsychological Assessment

All participants underwent clinical evaluation, performed by a research nurse, and the Hong Kong version of the Montreal Cognitive Assessment test (HK-MoCA) [21], performed by a trained research assistant.

### 2.3. MRI Acquisition

MRI scans were performed using a 3 Tesla MRI scanner (Achieva TX, Philips, The Netherland) with a standard 32-channel head coil. Structural images were acquired using 3D T1-weighted MPRAGE in the sagittal orientation with repetition time (TR)/echo time (TE)/inversion time = 6.8/3.2/847.9 ms, flip angle = 8°, field of view (FOV) = 256 × 240 × 204 mm^3^, and image resolution = 1 × 1 × 1.2 mm^3^. 3D FLAIR images were obtained with TR/TE = 4800/267 ms, thickness = 1.2 mm, FOV = 250 × 250 × 185 mm^3^, and image resolution = 1.2 × 1.2 × 1.12 mm^3^. DTI images were acquired with a spin-echo echo-planar sequence with TR/TE = 3900/81 ms, FOV = 230 × 230 mm^2^, image resolution = 3 × 3.1 mm^2^ (slice thickness of 3 mm), b-values of 0 and 1000 s/mm^2^, and 15 diffusion gradient directions. rs-fMRI images were obtained with a gradient-echo echo-planar sequence with TR/TE = 2000/30 ms, flip angle = 90°, FOV = 230 × 230 × 144 (mm), image resolution = 3.28 × 3.28 mm^2^, slice thickness = 4 mm, and number of volume = 180. During the rs-fMRI, participants were instructed to focus on a cross in the mirror and not think about anything.

### 2.4. PET-CT Acquisition

Patients with MCI or AD (*n* = 47) were required to fast for at least 6 h before the ^18^F-flutemetamol PET-CT scan. A bolus of ^18^F-flutemetamol at a dose of 185 MBq (nearly 5 mCi) was administered intravenously within 40 s. The scans were performed 90 min after the injection using an integrated in-line PET/CT scanner with 3D list mode. The duration of the scan was 30 min. Images were reconstructed using filtered back-projection with a slice thickness = 2~4 mm, matrix size = 128 × 128, and pixel size = 2 mm, with a full-width half-maximum (FWHM) post-smoothing filter with a Gaussian kernel of 5 mm.

### 2.5. White Matter Lesion Quantification

FLAIR images were used to quantify subcortical and periventricular white matter lesions using the Fazekas scale [22] by a trained scientist and were subsequently verified by an experienced neuroradiologist.

### 2.6. Dementia/Cognitively Impaired Subtype Classification

The diagnosis of cognitive impairment was determined by the consensus between a neuroradiologist (HKFM) and two geriatricians (YFS, PC, or JSKK) based on the findings from clinical (baseline and follow-up) and neuropsychological (HK-MoCA) evaluations, ^18^F-flutemetamol PET-CT scans, structural MRI, MRA, and ASL [20]. The diagnoses of MCI were determined using the clinical criteria from [23] and those of AD from [24] together with a positive Aβ diagnosis.

### 2.7. Amyloid Burden

The visual assessment of ^18^F-flutemetamol PET-CT scans was performed to determine whether an MCI patient was Aβ-positive or negative according to the criteria used in previous studies [25,26]. The assessment was performed by an experienced neuroradiologist (HKFM) who had successfully completed an electronic training program developed by GE Healthcare for the interpretation of ^18^F-Flutemetamol images.

The post-processing procedure included realignment, co-registration, and normalization using semi-automatic commercially available software (Cortex ID software, GE Healthcare Ltd., Waukesha, WI, USA). Quantitative analysis of 16 regions of interest (ROIs) was made by the Cortex ID software, including bilateral prefrontal, anterior cingulate, precuneus/posterior cingulate, parietal, lateral temporal, occipital, sensorimotor, and mesial temporal regions. Standardized uptake values (SUVs) were calculated in all regions and normalized for the injected dose and body weight of each subject. The standardized uptake value ratio (SUVR) was defined as the ratio between two SUVs of different regions from within a single scan to avoid the bias of injected activity, using body weight and the volume to mass conversion factor, and was referenced to the pons in our data. The composite SUVR, representing the global Aβ burden, was calculated as the average SUVR value of the area-weighted mean for the 16 cortical ROIs. Cortex ID also offered regional z-scores as compared with a normal database for ^18^F-flutemetamol.

### 2.8. Network Construction

#### 2.8.1. Anatomical Parcellation

The entire brain was segmented into 90 regions (45 regions per hemisphere) using the automated anatomical labelling (AAL) template [27]. These regions were subsequently used as the nodes of the structural and functional connectivity networks for each subject.

#### 2.8.2. Structural Brain Network Construction

The DTI data were corrected for motion and eddy current geometric distortions and brain tissue extraction was performed using the fMRIB Software Library, Oxford, UK’ (FSL, http://fsl.fmrib.ox.ac.uk/fsl, accessed on 21 November 2020). Whole-brain tractography was conducted in the native space using the Fiber Assignment by Continuous Tracking algorithm from the Diffusion Toolkit (Athinoula A. Martinos Center for Biomedical Imaging, Massachusetts, MA, USA; http://trackvis.org/dtk/, accessed on 21 November 2020) with an FA threshold of 0.1 and an angle threshold of 35°. The brain parcellations from AAL in the standard Montreal Neurological Institute (MNI) space were warped to the individual’s native space by the inverse transformations of image normalization and co-registration using SPM12 (The Wellcome Centre for Human Neuroimaging, London, UK; https://www.fil.ion.ucl.ac.uk/spm/software/spm12/, accessed on 9 January 2020). The fiber number between different brain parcellations was obtained using the UCLA Multimodal Connectivity Package (Center for Cognitive Neuroscience, University of California, Los Angeles, CA, USA; https://www.ccn.ucla.edu/wiki/index.php, accessed on 8 December 2020). A 90 × 90 structural connectivity (SC) matrix was obtained for subsequent analyses.

#### 2.8.3. Functional Brain Network Construction

The analysis of the rs-fMRI data was performed using the DPABI toolbox (State Key Laboratory of Cognitive Neuroscience and Learning & IDG/McGovern Institute for Brain Research, Beijing Normal University, Beijing, China; http://rfmri.org/dpabi, accessed on 9 January 2020) based on SPM12. The first ten dynamics were discarded, and the slices were corrected for different signal acquisition times. Next, the rs-fMRI data were realigned using a six-parameter (rigid body) linear transformation with a two-pass procedure (registered to the first volume and then registered to the mean of all volumes after the first realignment). Subjects with head movements more than 3 mm in any direction or over 3° were excluded in subsequent analysis. Tissue maps were obtained from T1 structural images. The Diffeomorphic Anatomical Registration Through Exponentiated Lie algebra (DARTEL) tool [28] was used to normalize the structural images and tissue maps to the MNI space. Several nuisance signals, including Friston 24 head motion parameters [29] derived from realignment, and mean white matter and cerebrospinal fluid time series were regressed out from the time course in each voxel. All fMRI images were spatially normalized to the MNI space and resampled to 3 × 3 × 3 mm^3^ using the transformation parameters from DARTEL. The rs-fMRI data were then band-pass filtered (0.01 < frequency < 0.1 Hz) to reduce high-frequency components from respiratory and cardiac motion and low-frequency drift. Linear trends were also removed. Brain regional time series was subsequently obtained by averaging the rs-fMRI signal of the voxels of each brain parcellation from AAL. The 90 × 90 functional connectivity (FC) matrix was subsequently obtained by correlation between the time series of all pairs of brain regions. Fisher z-transformation was applied to transform the correlation coefficients. FC matrix entry with a negative correlation coefficient was set as zero [30].

### 2.9. The Structural–Functional Connectivity Coupling

To estimate the coupling between the structural and functional brain networks, the following procedures were performed: (1) brain connections without any structural connections were discarded; (2) the distribution of structural connections was rescaled to a Gaussian distribution [12]; and (3) Pearson correlation was performed between the entire SC and FC matrices. The resulting correlation coefficient was regarded as the coupling of the two networks, denoted as SC-FC coupling from hereon.

### 2.10. Statistical Analysis

All statistical analyses were performed using SPSS (SPSS Inc., Chicago, IL, USA). Brown–Forsythe ANOVA with Tukey’s post hoc test was performed for group statistics. Sex was tested using Pearson’s Chi-square test. The relationships between Aβ deposition and SC-FC coupling and between Aβ deposition and MoCA scores were estimated using partial Pearson correlation with age, sex, and Fazekas score as covariates. The correlation was performed separately for the cognitively impaired subjects with positive and negative Aβ deposition. A significance level of *p* < 0.05 was set for all statistical tests.

## 3. Results

A total of 61 subjects, including 47 patients (MCI_Aβ-: *n* = 21; MCI_Aβ+: *n* = 12; AD: *n* = 14) and 14 controls, were recruited. Two controls, one MCI_Aβ+, and two AD patients were excluded due to head motion during the rs-fMRI and DTI scans. The demographic and clinical characteristics of these subjects are summarized in Table 1. HC were significantly younger (*p* < 0.001; age: 54 ± 16.8 years old) than the patient cohorts. MCI_Aβ- (MoCA: 23.0 ± 3.2; Fazekas scale: 3.95 ± 1.28), MCI_Aβ+ (MoCA: 19.0 ± 4.3; Fazekas scale: 3.64 ± 1.80), and AD (MoCA: 12.0 ± 7.6; Fazekas scale: 3.00 ± 1.65) patients had significantly (*p* < 0.001) lower MoCA and higher Fazekas scores than HC (MoCA: 28.7 ± 1.3; Fazekas scale: 0.58 ± 0.67), with MCI_ Aβ- patients having significantly (*p* = 0.004) higher MoCA scores than AD patients. MCI_ Aβ+ (0.71 ± 0.12) and AD (0.78 ± 0.10) patients had a significantly (*p* < 0.001) higher Aβ deposition when compared with MCI_ Aβ- (0.42 ± 0.04) patients.

**Figure 1:**
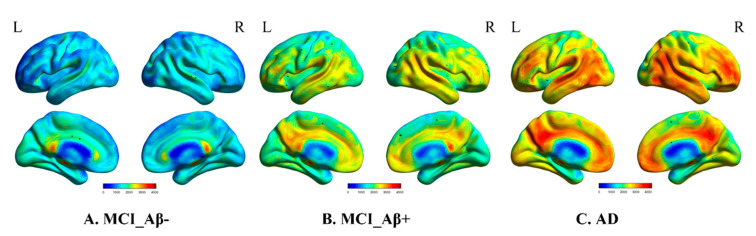
^18^F-flutemetamol PET-CT images of patients with (**A**) MCI_Aβ-, (**B**) MCI_Aβ+, and (**C**) AD. HC: healthy controls; Aβ: amyloid beta; MCI_Aβ-: mild cognitive impairment patient with negative Aβ; MCI_Aβ+: mild cognitive impairment patient with positive Aβ; AD: Alzheimer’s disease.

Figure 1 shows the group average of ^18^F-flutemetamol PET-CT images from the MCI_Aβ-, MCI_Aβ+, and AD cohorts.

**Figure 2:**
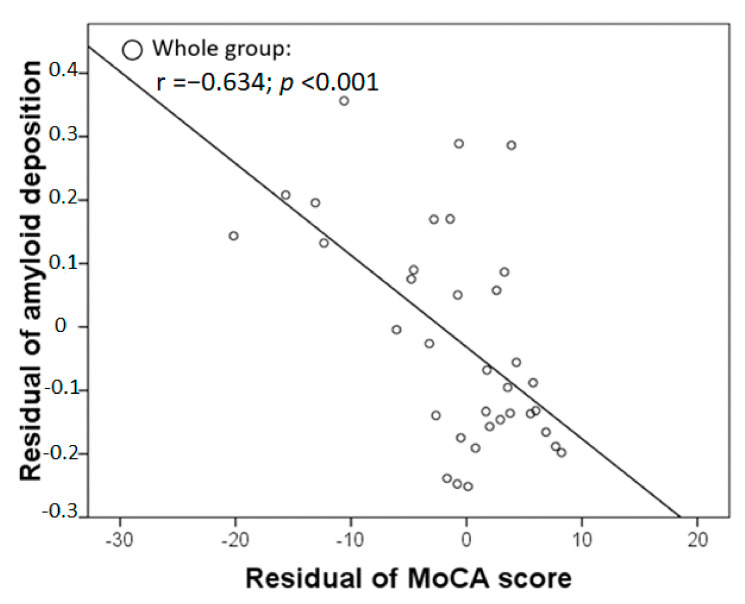
Scatter plot between MoCA score and the whole-brain Aβ deposition (partial Pearson correlation controlling for age, sex and Fazekas score). Aβ: amyloid beta; MoCA: Montreal Cognitive Assessment.

After controlling for age, sex, and Fazekas score, a significant negative correlation (r = −0.634, *p* < 0.001) between MoCA scores and whole-brain Aβ deposition was observed (Figure 2).

**Figure 3:**
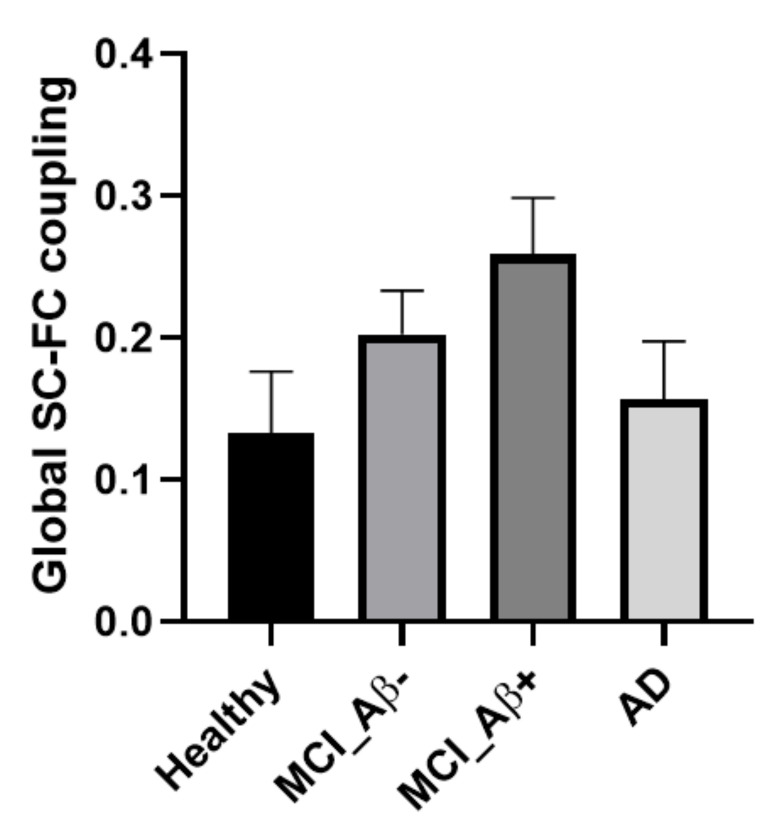
Group comparison of global coupling between structural and functional brain networks (SC-FC coupling) for different cohorts. Note that there was no significant difference between groups (*p*-value = 0.16; one-way Brown–Forsythe ANOVA).

Table 1 and Figure 3 illustrate the SC-FC coupling of different patient cohorts.

**Figure 4:**
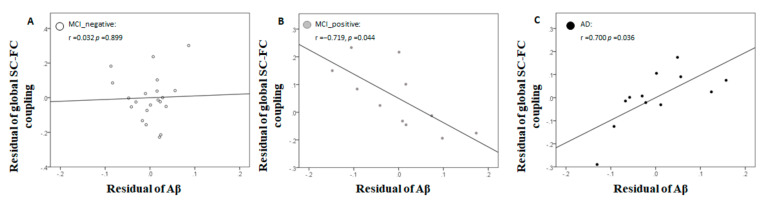
Scatter plots between Aβ deposition and global SC-FC coupling after controlling for age, sex, and Fazekas score for (**A**) MCI_Aβ-, (**B**) MCI_Aβ+, and (**C**) AD patients. Partial Pearson correlation controlling for age, sex, and Fazekas score was performed.

There was no significant difference in global SC-FC coupling between groups (*p* = 0.16); however, an increasing trend in HC to MCI_Aβ+ patients was observed. After adjusting for age, sex, and Fazekas score, there was a significant negative correlation between Aβ deposition and the SC-FC coupling (r = −0.719, *p* = 0.044; Figure 4B) for MCI_Aβ+ patients, and a significant positive correlation for AD patients (r = 0.700, *p* = 0.036; Figure 4C), but no significant correlation for MCI_Aβ- patients (r = 0.032, *p* = 0.899, Figure 4A).

## 4. Discussion

The effect of amyloid-β along the Alzheimer’s disease continuum was investigated using the global coupling between structural and functional brain networks. Although there was no difference in group-level global SC-FC coupling between different patient cohorts, Aβ pathology had distinctive stage-specific effects on the global coupling, consistent with previous studies that have investigated the structural and functional brain networks of AD separately [11].

### 4.1. Prodromal Alzheimer’s Disease

Our results show that, during the prodromal stage of AD, the effect of Aβ pathology on global SC-FC coupling was markedly different in that significant negative association was only observed for MCI_Aβ+ patients (Figure 4B) but not MCI_Aβ- patients (Figure 4A). These results may indicate that the global coupling between the structural and functional brain networks of MCI_Aβ- patients could be resilient to Aβ pathology during the early phase of Aβ accumulation. This may be attributable to the relatively focal effect of Aβ, as it initially preferentially accumulates only in a few selected locations of the brain, primarily the default mode network [4].

When the Aβ level became abnormal, the global SC-FC coupling of MCI_Aβ+ patients decreased with the level of Aβ pathology (Figure 4B) and showed an increasing group-level trend compared to HC (Figure 3). Previous studies have demonstrated that abnormality in functional connectivity during this phase of AD progression was correlated with the levels of Aβ pathology, but not tau pathology, in preclinical and prodromal AD [31,32]; this association first occurred in the default mode network in preclinical AD, before gradually spreading to other brain networks over the AD continuum [33]. Apart from the functional brain network, the association between the topology of the structural brain network and the Aβ burden for patients with MCI [34] along with a diffuse loss of structural connectivity over the course of Aβ accumulation was also observed [35,36]. Taken together, the progressive effect of Aβ pathology up to the prodromal stage of AD not only affects structural and functional brain networks individually, but also the global coupling between the two networks.

It is of note that the global coupling of MCI_Aβ+ patients decreased as the Aβ burden increased amidst an increasing group-level trend compared to HC. According to a study of regional SC-FC coupling by Gu et al., lower-order brain regions, such as those in the visual and subcortical networks, tend to have higher regional coupling, likely suggesting that the direct structural connections underlying these brain regions serve as the main relay for propagation of brain signals [37]. On the other hand, the higher-order cortices, such as those in the default mode network, tend to have lower regional coupling, likely suggesting that the indirect structural connections underlying these cortical areas may be more important for signal relay [37]. Taken together, the negative association between the Aβ pathology and global SC-FC coupling of MCI_Aβ+ patients may indicate that the direct structural connections between different regions across the brain gradually cannot constrain and facilitate the underlying brain signal propagation as Aβ burden increases.

### 4.2. Alzheimer’s Disease Dementia

Our results show a contrasting effect of Aβ pathology on the global SC-FC coupling of patients with MCI_Aβ+ (Figure 4B) versus those with AD (Figure 4C), despite their similar level of Aβ burden. Previous studies have shown that Aβ accumulation diffusely spreads across the cerebral cortex in the transition from prodromal AD to AD [38,39]. More importantly, the functional connectivity of the hub of multiple functional brain networks was shown to be associated with Aβ pathology [40], and that Aβ accumulation was colocalized with these abnormal functional connectivities for patients with AD [41]. Taken together, the fact that the effect of Aβ pathology spreads from non-hub brain regions to hub regions in the functional brain network during the AD dementia stage may underpin the contrasting effect on global SC-FC coupling observed in patients with MCI_Aβ+ versus those with AD. Of note is that the interpretation of our results may be confounded by the interactive effects of Aβ and tau on functional connectivity [42].

### 4.3. Previous Investigations of Brain Network Coupling in Alzheimer’s Disease

In a study of 38 normal controls, 40 MCI patients, and 19 AD patients, the SC-FC coupling of all connections of MCI and AD patients was enhanced, but no difference was found in the rich club connections compared to normal controls [43]. Another study on patients with mild cognitive impairment with no dementia (CIND, similar to MCI; *n* = 61) and moderate CIND (*n* = 56) demonstrated that moderate CIND patients showed higher global SC-FC coupling than healthy older subjects [44]. Another study showed no significant difference in global SC-FC coupling between AD patients and healthy controls, as well as the increased coupling of the default mode network of AD patients [45]. On the contrary, Sun et al. reported a significant decrease in the global SC-FC coupling of AD patients (*n* = 12) compared to HC (*n* = 14) and amnestic MCI patients (*n* = 15) [18]. Together with the results of our study showing no significant group difference in global SC-FC coupling, the inconsistency in these findings may be the result of the variations in the methods used for brain network construction and diagnostic criteria of MCI and AD.

### 4.4. Limitations

Firstly, because tau is another hallmark pathology of AD [42,46], its effect on the SC-FC coupling warrants further investigation. In particular, tau accumulation was demonstrated to be associated with functional connectivity regardless of the presence of Aβ deposition and dementia symptoms [47]. The investigation of the effect of tau on global coupling may thus potentially provide new knowledge on how to disentangle the contrasting effect of Aβ on the global coupling of patients with MCI_Aβ+ versus those with AD. Secondly, previous studies have demonstrated that several factors, such as genes, age, and cognitive reserve (CR), could modify the progression from prodromal AD to AD [6]. In particular, CR could influence the trajectory of AD progression [48]. It would therefore be worthwhile to investigate whether the association between Aβ pathology and global coupling along the AD continuum would change for patients with high CR versus those with low CR. Thirdly, our DTI data were acquired along 15 diffusion-encoding directions to reduce the total scan time, thereby increasing the bias in the estimation of white matter tracts.

## 5. Conclusions

We successfully demonstrated that Aβ pathology has distinctive stage-specific effects on the global coupling between the structural and functional brain networks.

## Figures and Tables

**Table 1:** Demographic and clinical characteristics of healthy controls and patients with mild cognitive impairment or Alzheimer’s disease.

	HC	MCI_Aβ-	MCI_Aβ+	AD	*p*-Value
Final sample size (*n*) ^^^	12	21	11	12	-
Age range	54.0 ± 16.8 ^a,b,c^	75.9 ± 7.0 ^a^	74.5 ± 7.6 ^b^	74.5 ± 8.7 ^c^	<0.001
Sex (female/male)	7/5	11/10	7/4	7/5	0.94
HK-MoCA	28.7 ± 1.3 ^d,e,f^ (*n* = 12)	23.0 ± 3.2 ^d,g^ (*n* = 19)	19.0 ± 4.3 ^e^ (*n* = 7)	12.0 ± 7.6 ^fg^ (*n* = 9)	<0.001
Fazekas Scale	0.58 ± 0.67 ^h,i,j^	3.95 ± 1.28 ^h^	3.64 ± 1.80 ^i^	3.00 ± 1.65 ^j^	<0.001
Aβ deposition	-	0.42 ± 0.04 ^k,l^	0.71 ± 0.12 ^k^	0.78 ± 0.10 ^l^	<0.001
Global SC-FC coupling	0.13 ± 0.04	0.20 ± 0.03	0.26 ± 0.04	0.16 ± 0.04	0.16

Brown–Forsythe ANOVA with post hoc test: ^c,g,j^ *p* < 0.05; ^a,b,d,e,f,h,i,k,l^ *p* < 0.01 ^^^ after excluding subjects with large head motions. HC: healthy controls; Aβ: amyloid beta; MCI_Aβ-: mild cognitive impairment patient with negative Aβ; MCI_Aβ+: mild cognitive impairment patient with positive Aβ; AD: Alzheimer’s disease; SC-FC: structural-functional connectivity.

## Data Availability

The data presented in this study are available on request from the corresponding author.
